# Hsa_circ_0011385 accelerates the progression of thyroid cancer by targeting miR-361-3p

**DOI:** 10.1186/s12935-020-1120-7

**Published:** 2020-02-13

**Authors:** Fada Xia, Yong Chen, Bo Jiang, Ning Bai, Xinying Li

**Affiliations:** Department of General Surgery, Xiangya Hospital, Central South University, 87 Xiangya Road, Changsha, 410008 Hunan People’s Republic of China

**Keywords:** Thyroid cancer, hsa_circ_0011385, miR-361-3p, Migration and invasion, Apoptosis

## Abstract

**Background:**

Thyroid cancer is an endocrine malignancy that is growing in incidence worldwide. Despite progress in diagnostics and treatment of thyroid cancer, prognosis remains poor. Emerging research has shown that circular RNAs (circRNAs) have crucial regulatory roles in cancers. However, the possible functions and mechanisms of hsa_circ_0011385 remain undetermined.

**Materials and methods:**

Expression levels of hsa_circ_0011385 and miR-361-3p were evaluated by qRT-PCR assay. The interaction between hsa_circ_0011385 and miR-361-3p was verified by dual-luciferase reporter assay. Effects of hsa_circ_0011385 or miR-361-3p on cell viability, proliferation, cell cycle, apoptosis, migration and invasion were confirmed by cell counting kit-8 (CCK-8), carboxyfluoresceinsuccinimidyl ester (CFSE), flow cytometry, and Transwell assays in vitro. The effect of hsa_circ_0011385 on thyroid cancer progression was also determined by in vivo tumor formation assay. Target genes of miR-361-3p were predicted by gene ontology (GO) and Kyoto Encyclopedia of Genes and Genomes (KEGG) analysis, and the expression of apoptosis- and metastasis-related proteins were assessed by Western blot assay.

**Results:**

Hsa_circ_0011385 upregulated in thyroid cancer; hsa_circ_0011385 knockdown inhibited thyroid cancer cell proliferation, migration and invasion, and promoted cell cycle arrest and apoptosis. In addition, hsa_circ_0011385 could negatively regulate miR-361-3p by functioning as a sponge. Hsa_circ_0011385 promoted thyroid cancer cell proliferation, migration and invasion and suppressed cell cycle arrest and apoptosis by targeting miR-361-3p in vitro. We also found that hsa_circ_0011385 knockdown dramatically inhibited thyroid cancer growth in vivo. Furthermore, hsa_circ_0011385 regulated expression of apoptosis and metastasis-related proteins in thyroid cancer.

**Conclusions:**

Hsa_circ_0011385facilitated thyroid cancer cell proliferation, invasion and migration, and inhibited thyroid cancer cell cycle arrest and apoptosis by targeting miR-361-3p, suggesting that the hsa_circ_0011385/miR-361-3p axis might be a promising therapeutic target for thyroid cancer.

## Background

Cancer is one of the leading causes of death in humans. Not surprisingly, carcinogenesis, cancer development, invasion, metastasis, and therapeutics of cancer have been a hot area of medical research [1]. Thyroid cancer is the most common cancer of the endocrine system [2]. In recent years, the prevalence of thyroid cancer has grown more rapidly than other malignant cancers and is higher in females than in males [3]. Thyroid cancer can be classified into thyroid papillary carcinoma, thyroid follicular carcinoma, thyroid anaplastic carcinoma originating from thyroid follicular epithelium, and thyroid medullary carcinoma originating from par follicular cells. Among the subtypes of thyroid cancer, thyroid papillary carcinoma and thyroid follicular carcinoma are traditionally defined as the differentiated thyroid cancers [4]. Their incidence rates are higher than 90%, their disease progression is slower and the curative effect is quite good. While undifferentiated thyroid cancer is rare, its death rate is extremely high [5]. Therefore, elucidating the potential molecular mechanisms of thyroid cancer pathogenesis may contribute to the treatment of thyroid cancer patients.

Non-coding RNAs (ncRNAs) mainly include microRNAs (miRNAs), long non-coding RNAs (lncRNAs), and circular RNAs (circRNAs) [6, 7]. Emerging research shows that ncRNAs are important gene regulators in a variety of diseases [8, 9]. In recent years, circular RNAs (circRNAs) have received much more attention. circRNAs are formed by the head-to-tail splicing of several exons of target gene [10] and usually manifest as covalently closed loop structures without 5′ caps and 3′ tails because of their specific formation [11]. At present, it is known that a large amount of circRNAs are differentially expressed in various diseases [12], such as cancers [13], cardiovascular disease [14], neurologic disease [15] and so on. Moreover, a growing number of studies have reported that circRNAs are deregulated in thyroid cancer [16, 17]. However, the possible functions and mechanisms of hsa_circ_0011385 in thyroid cancer remain unclear.

MicroRNAs (miRNAs) are a class of short RNA molecule with 20–22 nucleotides that can modulate gene expression by binding to the 3′-untranslated regions (3′-UTRs) of the targeted gene [18]. Abnormal expression of miRNAs has been identified in various diseases, such as neurodegenerative disorders, cancer, and inflammatory disorders [19–21]. Recent research has shown that miR-361-3p plays an essential role in cancers [22–24]. However, the molecular functions and mechanisms of miR-361-3p in thyroid cancer are still not well understood. In addition, a growing number of studies exploring the role of circRNAs in the pathogenesis of human cancers has shown that the circRNAs-miRNAs-mRNAs axis has indispensable effects [25, 26]. We found that there were binding sites between hsa_circ_0011385 and miR-361-3p by biological information. Therefore, we assumed that hsa_circ_0011385 may regulate miR-361-3p by acting as a sponge in thyroid cancer.

In this study, we explored the expression of hsa_circ_0011385 in thyroid cancer tissues and evaluated whether hsa_circ_0011385 could act as a sponge to negatively regulate miR-361-3p. In addition, we demonstrated the effects of hsa_circ_0011385 on thyroid cancer cell viability, proliferation, cell cycle, apoptosis, migration and invasion by miR-361-3p in vitro. We also evaluated the influence of hsa_circ_0011385 knockdown on thyroid cancer growth in vivo. Therefore, our study examined whether hsa_circ_0011385/miR-361-3pplays a critical role in thyroid cancer progression.

## Materials and methods

### Patient samples

Fifteen pairs of human thyroid cancer tissues and corresponding normal tissues were collected from patients who were diagnosed with thyroid cancer in the Xiangya Hospital, Central South University from 2014 to 2018. Written consent was obtained from all subjects. This study was approved by the Ethics Committee of the Hospital.

### Cell culture

Human papillary-thyroid-carcinoma BCPAP cells were purchased from Shanghai Academy of Sciences. Cells were all maintained in Dulbecco’s modified Eagle’s medium (DMEM), supplemented with penicillin (100 U/mL), streptomycin (100 μg/mL), and 10% fetal bovine serum (FBS, S Hyclone, Cat.No.SH30087.01), and maintained at 37 °C with 5% CO_2_ and 95% O_2_.

### Oligonucleotide transfection

The miR-361-3p mimics and hsa_circ_0011385 siRNAs as well as negative control (NC) were obtained from GenePharma (Shanghai, China). The BCPAP cells were transfected with miR-361-3p mimics, hsa_circ_0011385 siRNAs, or NC using Lipofectamine™ RNAi MAX (Invitrogen, Cat.No. 13778075) according to the manufacturer’s instructions.

### Vector construction and transduction

The full-length hsa_circ_0011385 cDNA was produced by PCR using the Primer STAR Max DNA Polymerase Mix (Takara, Japan). The primers were hsa_circ_0011385-KpnI-F: 5′-AGCTGGCCCTTCTCAAGACGGATCCGAAAGCAGCTGGCCCTTCTCAAG-3′, hsa_circ_00113851-XhoI-R: 5′-ACTATACTGGTTGAGCTCTCCACTCGAATTCCTTGGCACTATACTGGTTGAGCTCT-3′. The PCR products were then sub-cloned into the vector pcDNA3.1+ (Invitrogen) to establish the recombinant plasmid pcDNA-hsa_circ_0011385. The myocardial cells were transfected with pcDNA-hsa_circ_0011385or control (pcDNA3.1+) by using Lipofectamine RNAi MAX reagent (Invitrogen, Cat. No. 13778075) according to the manufacturer’s instructions.

### Fluorescence in situ hybridization (FISH) assay

In situ hybridization was carried out using probes specific to the hsa_circ_0011385 sequence. Biotin-labelled specific RNA probes were transcribed from hsa_circ_0011385 PCR fragments using the biotin-labelling mix and RNA polymerase (Roche, China) according to instructions provided by manufacturers. After growth to the exponential phase, MGC-803 cells were fixed using 4% formalin. Tissue were cut into 4 μm sections and then fixed with 4% formalin. Cells and tissues were then hybridized in hybridization buffer with biotin-labeled probes specific to hsa_circ_0011385. Signals were measured by tyramide-conjugated Alexa 488 fluorochrome tyramide signal amplification (TSA) kit. The results were viewed using a Laser Scanning Confocal Microscope (Leica, Germany).

### RNA extraction and quantitative real-time PCR (qRT-PCR) assay

Total RNAs of thyroid cancer tissues and the treated BCPAP cells were extracted by using TRIzol buffer (#9109, Takara, Japan). cDNA was synthesized with the Reverse Transcription kit (Takara, Japan). qRT-PCR assay was carried out with BestarTM qPCR Master Mix (#2043, DBI Bioscience, China) on ABI7300 system. The sequence of primers used in this study is shown in Table 1. The relative expression levels were analyzed using the 2^−ΔΔCt^ method [27].

**Table 1:** The sequences of primers for RT-qPCR assay

Gene	Primer sequences
GAPDH	Forward: 5′-TGTTCGTCATGGGTGTGAAC-3′ Reverse: 5′-ATGGCATGGACTGTGGTCAT-3′
hsa_circ_0011385	Forward: 5′-TGACAACAATGAGCCCTACA-3′ Reverse: 5′-TTTCCTTGGCACTATACTGG-3′
miR-361-3p	Forward:5′-ACACTCCAGCTGGGTCCCCCAGGTGTGATTCTG-3′ Reverse:5′-CTCAACTGGTGTCGTGGAGTCGGCAATTCAGTTGAGAAATCAGA -3′
U6	Forward: 5′-CGCTTCACGAATTTGCGTGTCAT-3′ Reverse: 5′-GCTTCGGCAGCACATATACTAAAAT-3′

### Western blot assay

Proteins were extracted using RIPA buffer (Beyotime, Shanghai, China). The concentration of proteins was determined by BCA kit (Thermo Scientific). Proteins (40 μg) were isolated by 8% SDS-PAGE, and transferred to polyvinyl fluoride (PVDF) membrane (PMILLIPORE, IPVH00010). The membranes were blocked with 5% nonfat dried milk (BD Biosciences), and then were incubated with primary antibodies overnight at 4 °C. The next day, membranes were incubated with horseradish peroxidase-conjugated secondary antibodies (1:2000, Southern Biotech, 4050-05) for 2 h. The membranes were treated with the enhanced chemiluminescent reagents (MILLIPORE, WBKLS0500). The signals were examined by medical X-ray film (XBT-1, Eastman Kodak Company, NY, and USA). The primary antibodies in the present study were glyceraldehyde 3-phosphate dehydrogenase (GAPDH; 1:1000, KC-5G5), anti-Bax (1:1000, Abcam; cat no. ab32503), anti-caspase-3 (1:100, Santa Cruz Biotechnology, cat no. sc-7148), anti-tissue inhibitors of metalloproteinase (anti-TIMP; 1:1000, Abcam, ab86482), anti-matrix metallopeptidase 2 (anti-MMP2; 1:1000, Abcam, ab37150), and anti-MMP9 (1:1000, Abcam, ab73734).

### Carboxyfluoresceinsuccinimidyl ester (CFSE) assay

The proliferation capacity was detected by CFSE dye (Life Technologies-Molecular Probes, Grand Island, NY, USA). The treated BCPAP cells were incubated with CFSE (10 nM) for 30 mins, and then washed with complete medium. The labeled cells were incubated with antibody for 4 days and analyzed by flow cytometry.

### Flow cytometry analysis

After transfection, BCPAPcells were collected and centrifuged for 10 mins with 2000 rpm. Cells were then washed with pre-chilled phosphate-buffered saline three times, fixed with 4% paraformaldehyde for 1 h, and then stained with the apoptosis detection kit (Biolegend, San Diego, CA, USA) or Presidium (PI) (Beyotime, Suzhou, China) respectively for 10 mins at room temperature. Finally, the cells were subjected to apoptosis and cell cycle analysis with a flow cytometer (FACSCanto™ II, BD Biosciences).

### Hoechest staining

To observe cell apoptosis visually, cells (5 × 10^4^ per well) underwent different treatments were seeded into 6-well plates and stained with Hoechst 33258 solution (Beyotime Company, Shanghai, China) for 5 min. Hoechst 33258 penetrates the plasma membrane and stains DNA blue fluorescent, thereby being used to observe the morphological changes of apoptosis [28, 29]. The fluorescence microscope was used to observe the morphological changes of cell nuclei.

### Tunel staining

Briefly, dry the cell slides naturally and then fixed with 4% paraformaldehyde for 15 min. Then blocked with 3% hydrogen peroxide and followed with 1% Triton X-100 to increases the cell membrane permeability. Then cells were staining with TdT-mediated dUTP nick end labeling (TUNEL) (Roche, 11684817910, USA) according to the instruction manual. The nucleus was stained with DAPI. Images were captured using a fluorescence microscope (Olympus, Japan).

### Migration and invasion analysis

Thyroid cancer cell invasion or migration were examined by Transwell assay with 8-µm pores chambers (Corning Incorporated, Corning, NY, USA), with or without Matrigel matrix (BD Biosciences, Franklin Lakes, NJ, USA), respectively. The treated BCPAP cells were harvested and re-suspended in culture medium, to a final concentration 1 × 10^4^ cells/mL. Subsequently, 0.2 mL of the thyroid cancer cell suspension solution was added to the upper chamber, and 0.5 mL culture medium containing 20% FBS was added to the lower chamber. After incubation at 37 °C for 24 h, the invaded and migrated thyroid cancer cells were stained with 0.5% crystal violet (Beyotime Institute of Biotechnology, Haimen, China) and quantitated under a microscope.

### Plasmid construction and dual luciferase activity assay

To establish the plasmids, the sequence of hsa_circ_0011385 containing wild type (WT) or mutated (MUT) potential binding site for miR-361-3p was inserted downstream of the luciferase gene in the luciferase vector psi-CHECK (Promega, Madison, USA).

The primers were hsa_circ_0011385-WT-F: 5′-CCGCTCGAGGAAAGCAGCTGGCCCTTCTCAA-3′, hsa_circ_0011385-WT-R: 5′-ATTTGCGGCCGCCTTGGCACTATACTGGTTGAGCTCTC-3′; hsa_circ_0011385-MUT-F: 5′-ACCAGTGCTGTTTGGATCTGAATGGGGGAGTGCATCA-3′, hsa_circ_0011385-MUT-R: 5′-TGATGCACTCCCCCATTCAGATCCAAACAGCACTGGT-3′. For dual luciferase activity assay, BCPAP cells were cultured in 24-well plates and then transfected with WT or Muthsa_circ_0011385 and miR-361-3p mimics or its negative control using Lipofectamine 2000 Reagent (Life Technologies, Thermo Fisher Scientific, Inc., Waltham, MA). After 48 h, luciferase activity was detected by a Dual-Luciferase Assay Kit (Promega, Madison, WI).

### Pathway enrichment analysis

The target genes of miR-361-3pwereanalyzed by Kyoto Encyclopedia of Genes and Genomes (KEGG) via the Database for Annotation, Visualization and Integrated Discovery (DAVID, https://david.ncifcrf.gov/).

### Gene ontology (GO) enrichment analysis

The Database for Annotation, Visualization and Integrated Discovery (DAVID, http://david.abcc.ncifcrf.gov/) is a bioinformatics enrichment web tool for researchers to facilitate the transition from data collection to biological analysis to gain comprehensive high-throughput gene functional annotation analysis [30].

### Haematoxylin and eosin (HE) staining

The tumor tissues from mice in each group were transected, fixed in 4% paraformaldehyde, and dehydrated with different concentrations of alcohol. Then, 5-μm sections were stained with H&E solution (H8070, Solarbio, China). The images were obtained by using a microscope (Nikon Eclipse Ci, Japan) at 400× magnification.

### Immunohistochemistry (IHC) analysis

Briefly, tissues were cut into 4-µm-thick sections from embedded blocks. After antigen retrieval treatment in 10 mM citrate buffer, the tissue sections were incubated with rabbit anti-human primary antibodies against Ki67 at 4 °C overnight, followed by incubation with horseradish peroxidase-conjugated goat anti-rabbit IgG. All tissue slides were evaluated by two independent pathologists.

### Tumor formation assay in vivo

BALB/c nude mice is an albino, immunodeficient inbred mouse strain. BALB/c nude mice serve as a general-purpose animal model, this strain is used extensively for hybridoma and monoclonal antibody production and are especially useful for research in cancer therapy and immunology. They are very sensitive to carcinogens, and can develop lung tumors, reticular neoplasms, renal tumors, and other cancers. Here, the eight-week old male BALB/c nude mice were used for the in vivo tumor formation assay. The animals were purchased from the Department of Laboratory Animal Science, Central South University, and housed in a SPF room temperature under 12 h/12 h light/dark cycle, a temperature of 23 ± 3 °C, a relative humidity of 55 ± 15%, and could free access to food and water. The animal protocols were approved by the Institutional Animal Care and Use Committee of Central South University and performed according to the ARRIVE guidelines [31]. The BALB/c nude mice were randomly divided into 3 groups (n = 5) as follows: (A) Blank group (the mice were subcutaneously injected with BCPAP cells); (B) NC group (the mice were subcutaneously injected with BCPAP cells that were transfected with NC siRNA); (C) siRNA group (the mice were subcutaneously injected with BCPAP cells that were transfected with hsa_circ_0011385 siRNAs). BCPAP cells that were stably transfected with hsa_circ_0011385 siRNAs or NC were collected and re-suspended in medium at a concentration of 1 × 10^6^ cells/mL. Then, 200 μL of cell suspension was injected into the right flank of nude mice. Tumor growth was detected at 0, 1, 2, 3, 4, 5, 6, 7 and 8 weeks after injection, and the volume of tumors was recorded as the length × width^2^ × 0.5. Eight weeks after injection, euthanasia the BALB/c nude mice with CO_2_ and imaged immediately, and then the tumors were collected.

### Statistical analysis

Experimental data were presented as mean ± standard error of the mean (SEM) and analyzed with Graghpad (Ver. Prism 7, GraphPad Prism Software, La Jolla, CA, USA). The results were analyzed using Student’s t test and one-way analysis of variance analysis as appropriate, and *P *< 0.05 was considered statistically significant.

## Results

### Hsa_circ_0011385 was highly expressed in thyroid cancer

**Fig. 1:**
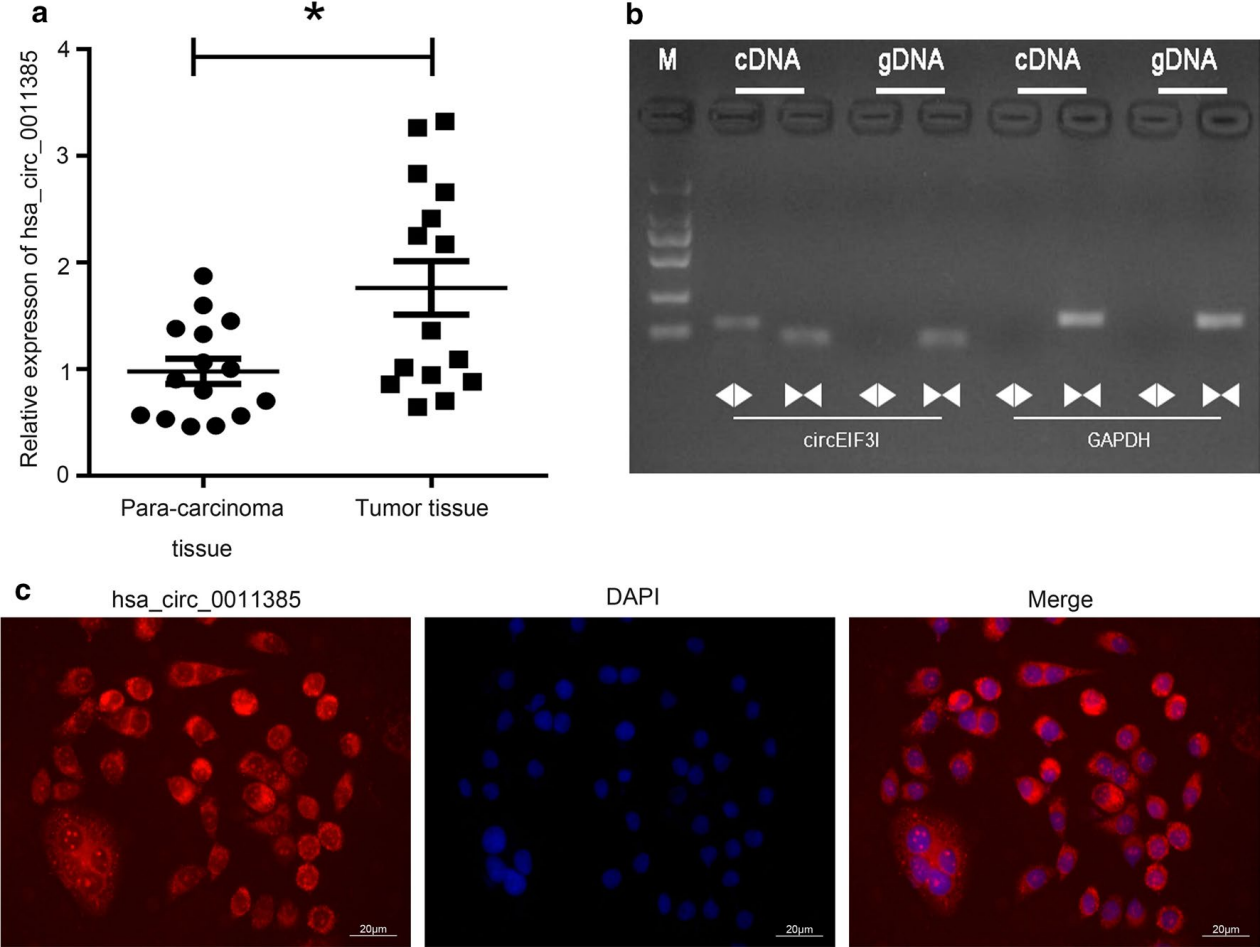
Hsa_circ_0011385 was highly expressed in thyroid cancer. **a** Expression of hsa_circ_0011385 was assessed by qRT-PCR assay in thyroid cancer tissues and corresponding para-carcinoma tissues (*P < 0.05). **b** The linear or circular EIF3I were amplified by PCR assay with divergent and convergent primers in thyroid cancer tissues. cDNA, complementary DNA; gDNA, genomic DNA. **c** The expression level of hsa_circ_0011385 was evaluated by FISH assay in BCPAP cells. Hsa_circ_0011385 was stained red and nuclei were stained blue using 4ʹ,6-diamidino-2-phenylindole (DAPI). Magnification, ×100. Scale bars, 50 µm

To explore the role of hsa_circ_0011385 in thyroid cancer, we examined its expression in thyroid cancer tissues by qRT-PCR assay. Results indicated that hsa_circ_0011385 expression was significantly higher in thyroid cancer tissues than in corresponding para-carcinoma tissues (*P *< 0.05, Fig. 1a). We then designed the convergent and divergent primers to amplify the linear EIF3I mRNA and hsa_circ_0011385 by using cDNA and gDNA extracted from thyroid cancer tissues. Results showed that hsa_circ_0011385 could only be amplified by divergent primers in cDNA of thyroid cancer tissues; EIF3I could be only amplified by convergent primers in cDNA and gDNA of thyroid cancer tissues, suggesting that hsa_circ_0011385 was indeed circular (Fig. 1b). In addition, we demonstrated that hsa_circ_0011385wasexpressed in BCPAP cells (Fig. 1c).

### Hsa_circ_0011385 knockdown inhibited thyroid cancer cell proliferation, and promoted cell cycle arrest

**Fig. 2:**
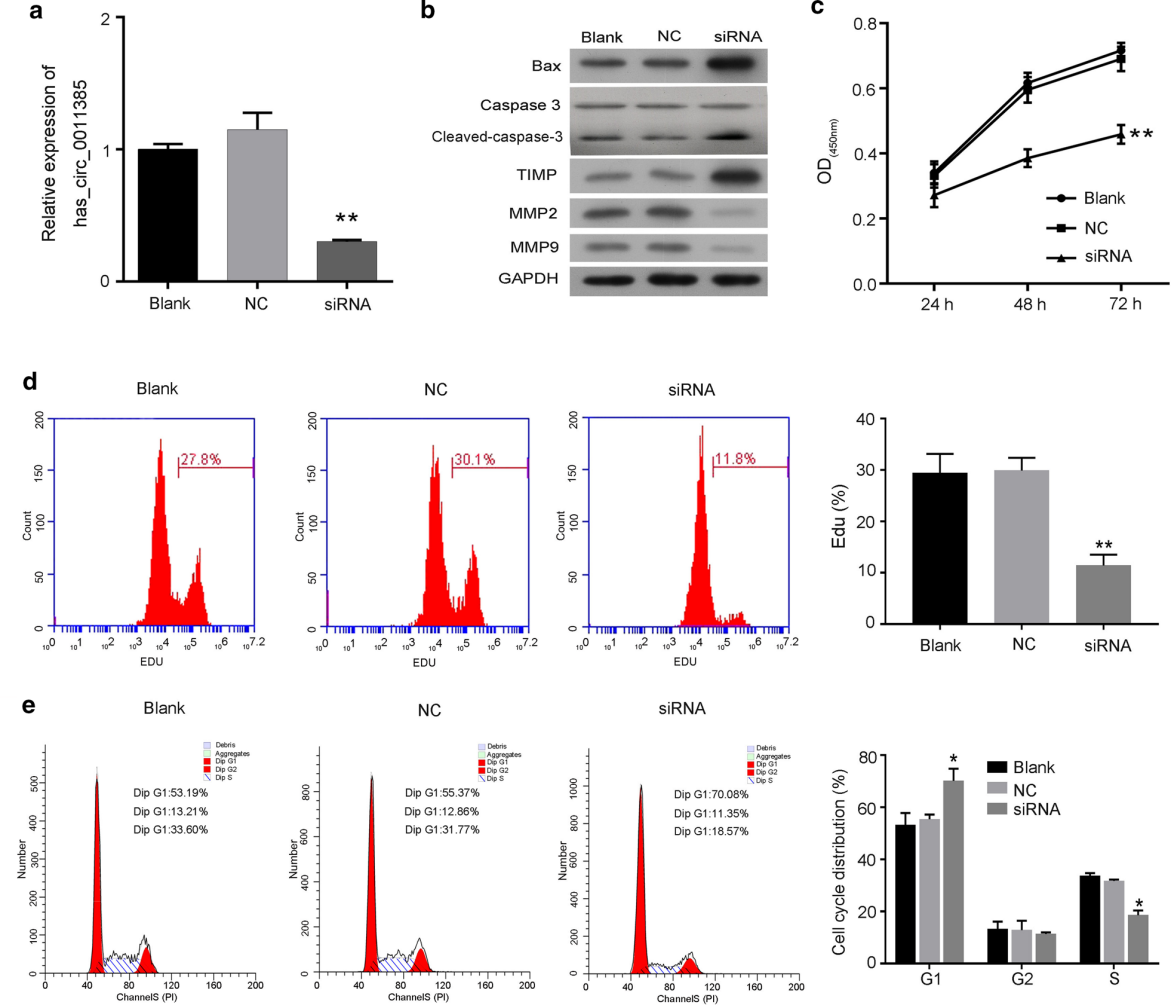
Hsa_circ_0011385 knockdown inhibited thyroid cancer cell proliferation and promoted cell cycle arrest. **a** BCPAP cells were transfected with hsa_circ_0011385 siRNAs, and the expression level of hsa_circ_0011385 was analyzed by qRT-PCR assay (**P < 0.01 vs. NC group). **b** Western blot assay was carried out to evaluate Bax, caspase-3, TIMP, MMP2 and MMP9 expression levels in BCPAP cells after hsa_circ_0011385 knockdown. GAPDH was used as a reference. **c** CCK-8 assay was performed to evaluate cell viability in BCPAP cells transfected with hsa_circ_0011385 siRNAs (**P < 0.01 vs. NC group). **d** Proliferation ability was assessed by CFSE assay in BCPAP cells after hsa_circ_0011385 knockdown (**P < 0.01 vs. NC group). **e** Cell cycle distribution was confirmed by flow cytometry in BCPAP cells after hsa_circ_0011385 knockdown (*P < 0.05 vs. NC group)

To determine whether hsa_circ_0011385could affect the progression of thyroid cancer, BCPAP cells were transfected with hsa_circ_0011385 siRNAs. The results from qRT-PCR assay revealed that hsa_circ_0011385 expression was dramatically decreased in the siRNA group compared with the NC group (*P *< 0.01, Fig. 2a). The protein expression levels of Bax, caspase-3 and TIMP were dramatically upregulated in the siRNA group relative to the NC group, whileMMP2 and MMP9 expression were significantly downregulated in the siRNA group compared to NC group (Fig. 2b). Subsequently, the effects of hsa_circ_0011385 knockdown on cell viability, proliferation, cell cycle arrest and apoptosis were assessed by CCK-8, CFSE and flow cytometry, respectively. CCK-8 results revealed that knockdown of hsa_circ_0011385 in BCPAP cells significantly reduced cell viability (*P *< 0.01, Fig. 2c), and the results from CFSE assay indicated that knockdown of hsa_circ_0011385 inhibited cell proliferation (*P *< 0.01, Fig. 2d). In addition, we found that compared with NC-transfected BCPAP cells, knockdown of hsa_circ_0011385significantly increased the G1 population (Blank, 53.19%; NC, 55.37%; siRNA, 70.08%) and reduced replication (S-phase) (Blank, 33.60%; NC, 31.77%; siRNA, 18.57%) in BCPAP cells (*P *< 0.05, Fig. 2e).

### Hsa_circ_0011385 knockdown accelerated thyroid cancer cell apoptosis, and inhibited cell migration and invasion

**Fig. 3:**
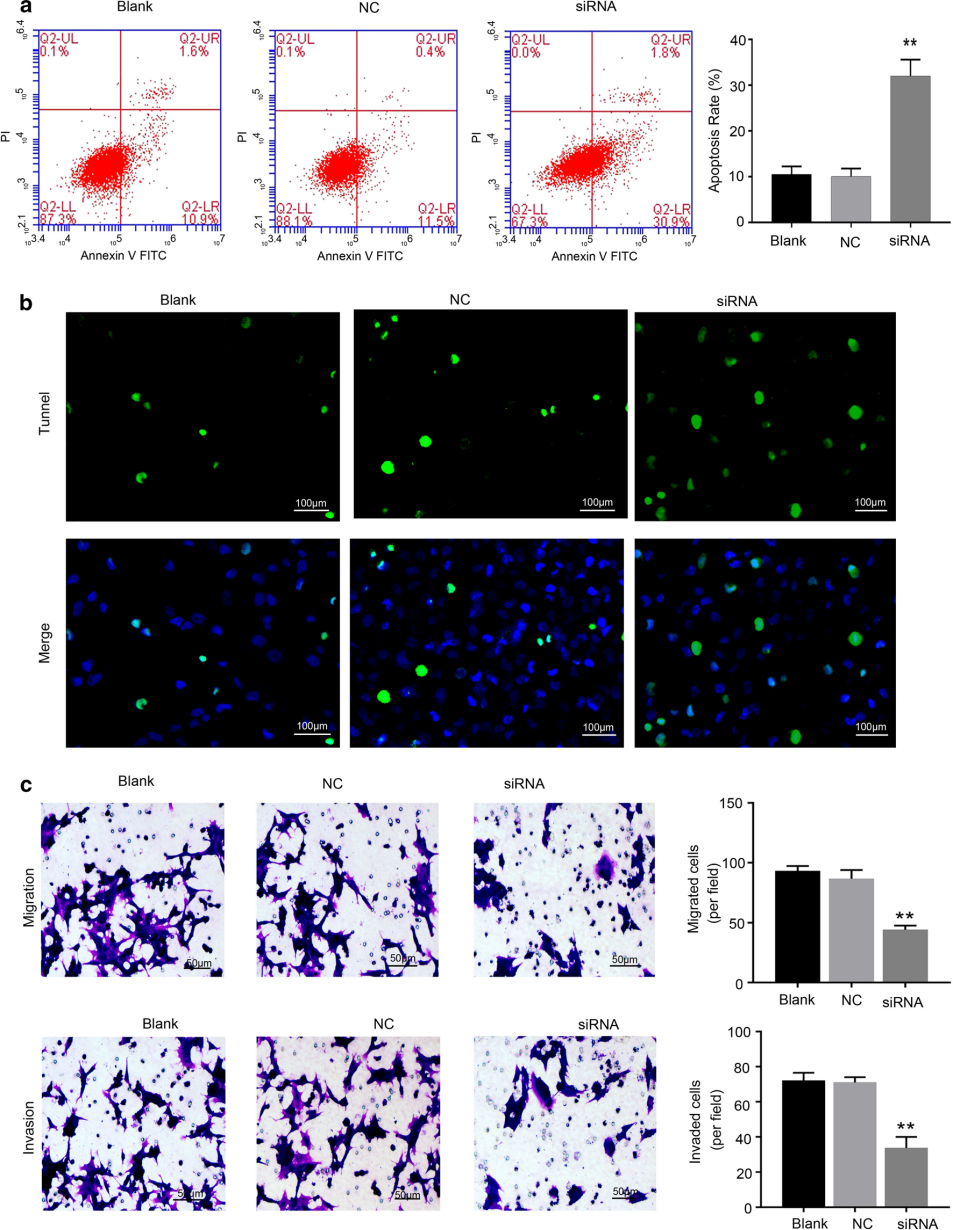
Hsa_circ_0011385 knockdown promoted thyroid cancer cell apoptosis and inhibited cell migration and invasion. **a** Effect of hsa_circ_0011385 knockdown on the apoptosis capacity of BCPAP cells was determined by flow cytometry (**P < 0.01 vs. NC group). **b** After hsa_circ_0011385 knockdown, the apoptosis ability of BCPAP cells was examined by Tunel staining, Magnification, ×200, Scale bars, 100 µm. **c** The migration and invasion capacities of BCPAP cells transfected with hsa_circ_0011385 siRNAs were assessed by Transwell assay (**P < 0.01 vs. NC group). Magnification, ×100. Scale bars, 50 µm

To determine whether hsa_circ_0011385inhibited BCPAP cell apoptosis and promoted migration and invasion, we knocked down hsa_circ_0011385 using siRNAs in BCPAP cells. The results from flow cytometryand Tunel staining indicated that knockdown of hsa_circ_0011385significantly increased the apoptotic capacity of BCPAP cells (*P *< 0.01, Fig. 3a, b). Hoechst staining also showed the similar results (Additional file 1: Figure S1A). In addition, we proved that BCPSP cells that were silenced by hsa_circ_0011385 showed a markedly attenuated migration and invasion abilities (*P *< 0.01, Fig. 3c).

### Hsa_circ_0011385 negatively regulated miR-361-3p

**Fig. 4:**
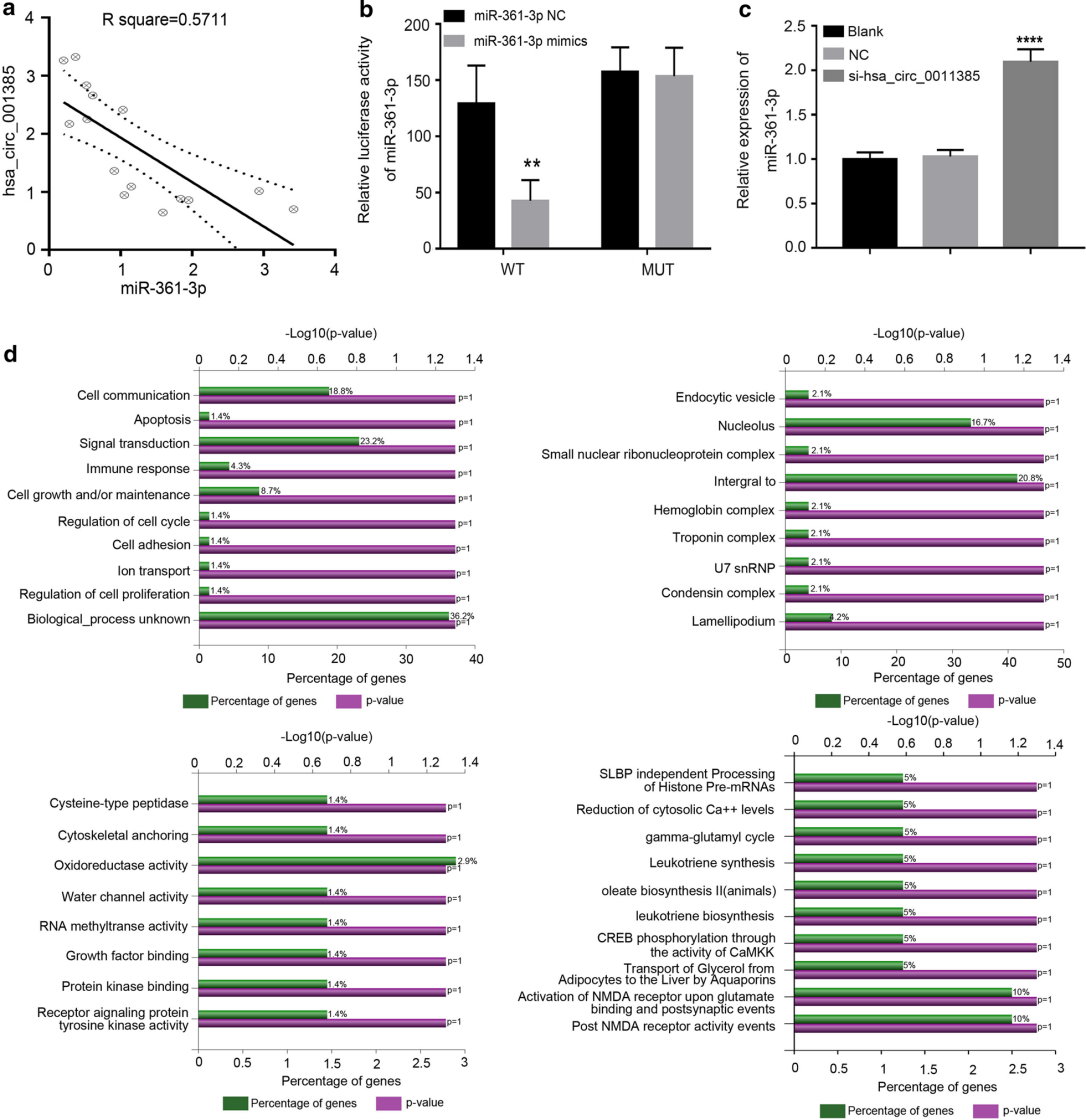
Hsa_circ_0011385 negatively regulated miR-361-3p and the Go annotations analysis and pathway analysis of the target genes. **a** Correlation between hsa_circ_0011385 and negatively regulated miR-361-3p expression in thyroid cancer tissues (*P *< 0.05, R^2^ = 0.5711). **b** A putative interactionsite with miR-361-3p in hsa_circ_0011385 was predicted. The interaction between hsa_circ_0011385 and miR-361-3p was verified by dual-luciferase reporter assay in BCPAP cells (**P < 0.01 vs. NC group). **c** Relative expression of miR-361-3p after qPCR detection of interference hsa_circ_0011385 (***P < 0.005 vs. NC group). **d** The percent of genes in GO term was shown in the bar chart of biological processes, cellular components, molecular functions and biological pathway

Previous studies indicated that circRNAs could act as a sponge for miRNAs. To further elucidate the underlying biological mechanisms of hsa_circ_0011385 in thyroid cancer, we explored the potential miRNAs associated with hsa_circ_0011385 by using miRBase prediction to identify potential miRNAs associated with hsa_circ_0011385, we found a binding site located between hsa_circ_0011385 and miR-361-3p. In addition, we examined the expression level of miR-361-3p in thyroid cancer tissues by qRT-PCR assay, and the results indicated that hsa_circ_0011385 expression was negatively correlated with miR-361-3p expression in thyroid cancer (*P *< 0.05, R^2^ = 0.5711, Fig. 4a). Luciferase reporter assay was also carried out to validate direct interaction between hsa_circ_0011385 and miR-361-3p in BCPAP cells, and the results showed that miR-361-3p mimics significantly attenuated the luciferase activity driven by wild type hsa_circ_0011385, but not the luciferase activity driven byMuthsa_circ_0011385 (*P *< 0.01, Fig. 4b). The hsa_circ_0011385 was interfered by siRNA, and qPCR results showed that miR-361-3p was significantly increased (*P* < 0.005, Fig. 4c).

### Go annotations analysis and pathway analysis of the target genes

To understand more about the roles of miR-361-3p, the differentially expressed genes were assigned to GO terms, including biological processes, cellular components and molecular function terms. As shown in Fig. 4d, the most enriched biological process terms were “cell communication”, “apoptosis”, and “signal transduction”. The most enriched cellular component terms were “endocytic vesicle membrane”, “nucleolus”, and “small nuclear rib nucleoprotein complex”. The most enriched molecular function terms were “cysteine-type peptidase activity”, “cytoskeletal anchoring activity” and “oxidoreductase activity”. In addition, the most enriched biological pathways were “SLBP independent processing of histone pre-mRNAs”, “reduction of cytosolic Ca^++^ levels”, and “gamma-glutamyl cycle”.

### Hsa_circ_0011385 promoted thyroid cancer cell proliferation and suppressed cell cycle arrest by miR-361-3p

**Fig. 5:**
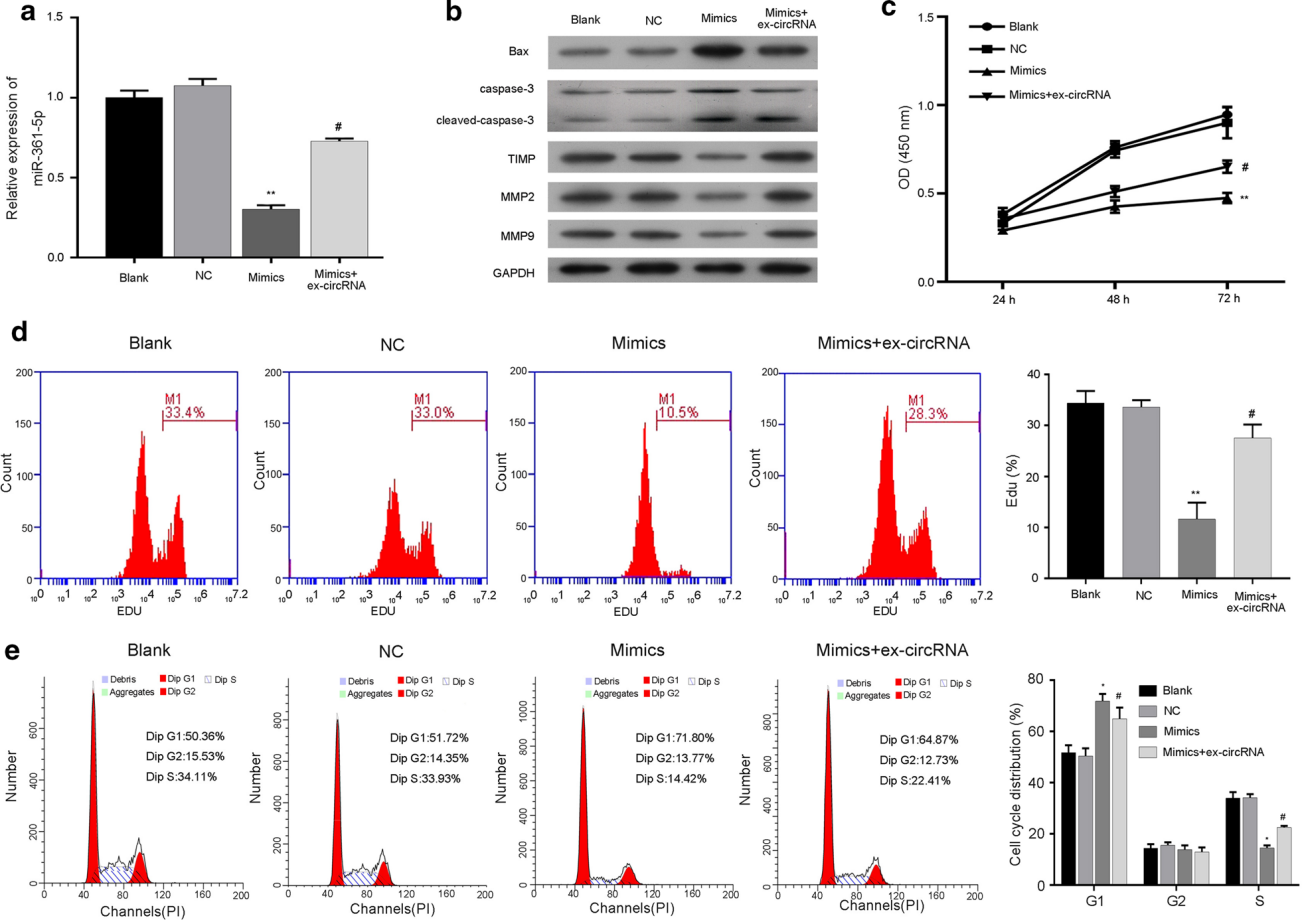
Hsa_circ_0011385 promoted thyroid cancer cell proliferation and suppressed cell cycle arrest by miR-361-3p. BCPAP cells were transfected with miR-361-3p mimics and/or hsa_circ_0011385 plasmid, respectively. **a** miR-361-3p expression was determined by qRT-PCR assay in each group (**P < 0.01 vs. NC group, ^#^*P *< 0.05 vs. mimics group). **b** Bax, caspase-3, TIMP, MMP2, and MMP9 expression levels were measured by Western blot assay in each group. GAPDH was used as an internal reference. **c** Cell viability was evaluated by CCK-8 assay in each group (**P < 0.01 vs. NC group, ^#^*P *< 0.05 vs. mimics group). **d** The proliferation abilities of BCPAP cells transfected with miR-361-3p mimics and/or hsa_circ_0011385 plasmid was detected by CFSE assay (**P < 0.01 vs. NC group, ^#^*P *< 0.05 vs. mimics group). **e** Flow cytometry was performed to examine the cell cycle distribution in each group (*P < 0.05 vs. NC group, ^#^*P *< 0.05 vs. mimics group)

We evaluated the effects of hsa_circ_0011385 on BCPAP cells by regulating miR-361-3p. Specifically, miR-361-3p mimics and hsa_circ_0011385 plasmid was transfected into BCPAP cells. qRT-PCR assay revealed that miR-361-3p expression was significantly downregulated in the miR-361-3p mimics group compared with the NC group, but was significantly upregulated in the miR-361-3p mimics plus hsa_circ_0011385group compared with themiR-361-3p mimics group (*P *< 0.05, *P *< 0.01, Fig. 5a). Western blot assay indicated that miR-361-3p increased Bax, caspase-3 and TIMP expression, while hsa_circ_0011385abolished the increase in BCPAP cells mediated by miR-361-3p mimics; miR-361-3p decreased MMP2, MMP9 expression, while hsa_circ_0011385 attenuated the decrease in BCPAP cells mediated by miR-361-3p mimics (Fig. 5b). Additional functional studies proved that miR-361-3p mimics were able to enhance the growth of BCPAP cells, while hsa_circ_0011385 abolished the enhancement, as shown by CCK-8 and CFSE assays (*P *< 0.05, *P *< 0.01, Fig. 5c, d). Meanwhile, miR-361-3p produced a significantly increased G1 population (Blank, 50.36%; NC, 51.72%; mimics, 71.80%), and a markedly decreased replication (S-phase) (Blank, 34.11%; NC, 33.93%; mimics, 14.42%) in BCPAP cells, while hsa_circ_0011385 attenuated the changes mediated by miR-361-3p mimics (*P *< 0.05, Fig. 5e). These results proved that hsa_circ_0011385 promoted the proliferation and suppressed cell cycle arrest of thyroid cancer cells through miR-361-3p.

### Hsa_circ_0011385 suppressed thyroid cancer cell apoptosis and facilitated migration and invasion by miR-361-3p

**Fig. 6:**
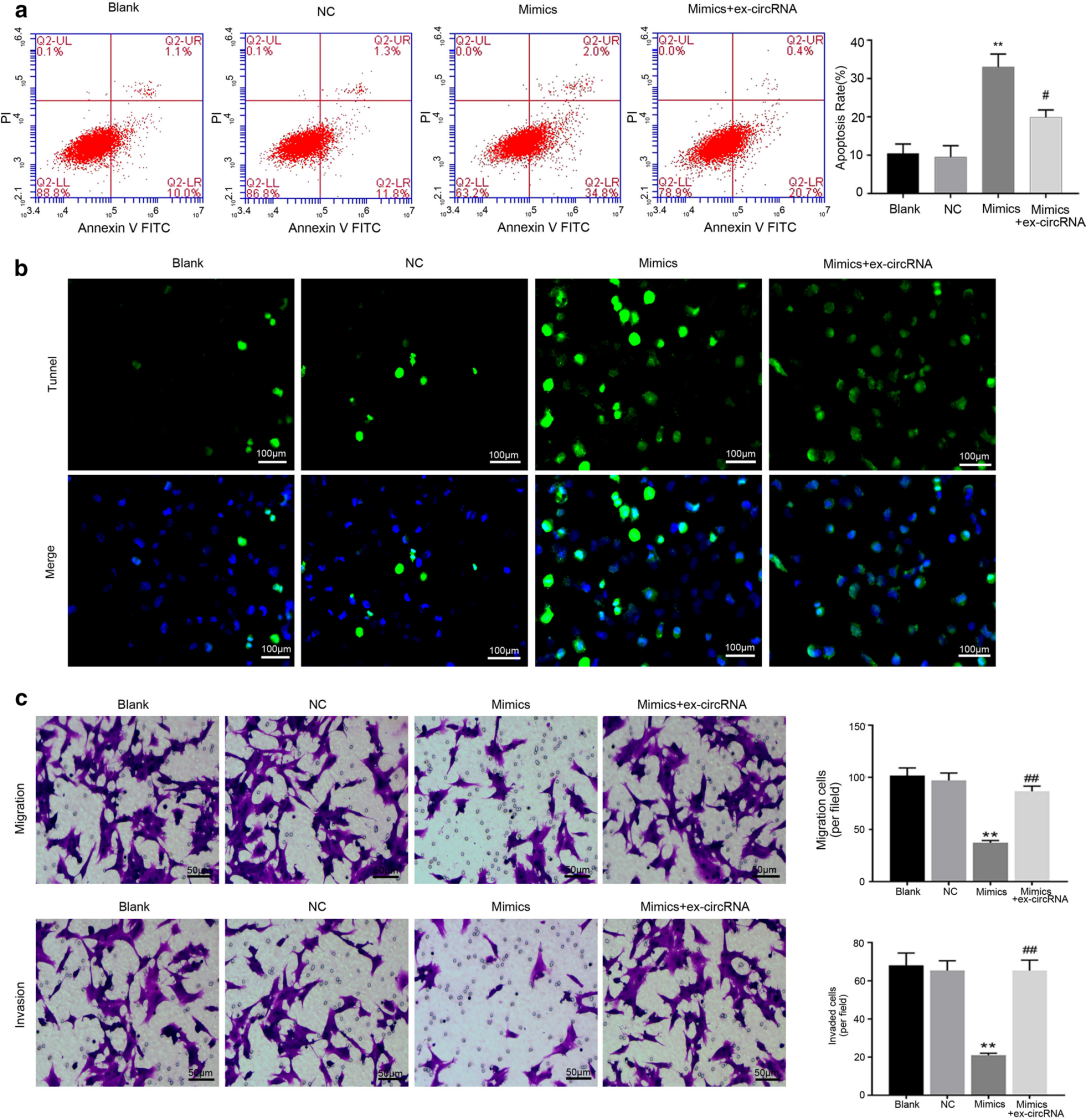
Hsa_circ_0011385 suppressed thyroid cancer cell apoptosis and facilitated cell migration and invasion by miR-361-3p. **a** The apoptosis capacity was determined by flow cytometryin BCPAP cells transfected with miR-361-3p mimics and/or hsa_circ_0011385 plasmid (**P < 0.01 vs. NC group, ^#^*P *< 0.05 vs. mimics group). **b** After transfection with miR-361-3p mimics and hsa_circ_0011385 plasmid, Tunel staining was utilized to examine the apoptosis ability of BCPAP cells. Magnification, ×200. Scale bars, 100 µm. **c** Transwell assay was carried out to assess the migration and invasion capacities of BCPAP cells transfected with miR-361-3p mimics and/or hsa_circ_0011385 plasmid (**P < 0.01 vs. NC group, ^##^*P *< 0.01 vs. mimics group). Magnification, ×100. Scale bars, 50 µm

In functional assays, flow cytometry and Tunel staining indicated that miR-361-3p significantly accelerated the apoptosis of BCPAP cells, while hsa_circ_0011385prevented the acceleration mediated by miR-361-3p mimics (*P *< 0.05, *P *< 0.01, Fig. 6a, b). Hoechst staining also showed the similar results (Additional file 1: Figure S1B). Transwell assay indicated that miR-361-3p significantly suppressed the migration and invasion capacities of BCPAP cells, and hsa_circ_0011385reversed the suppressive effects of miR-361-3p mimics on BCPAP cell migration and invasion abilities (*P *< 0.01, Fig. 6c). Therefore, these findings suggested that the effects of hsa_circ_0011385 on thyroid cancer cell apoptosis, migration and invasion specifically depend on miR-361-3p suppression.

### Hsa_circ_0011385 knockdown inhibited thyroid cancer growth in vivo

**Fig. 7:**
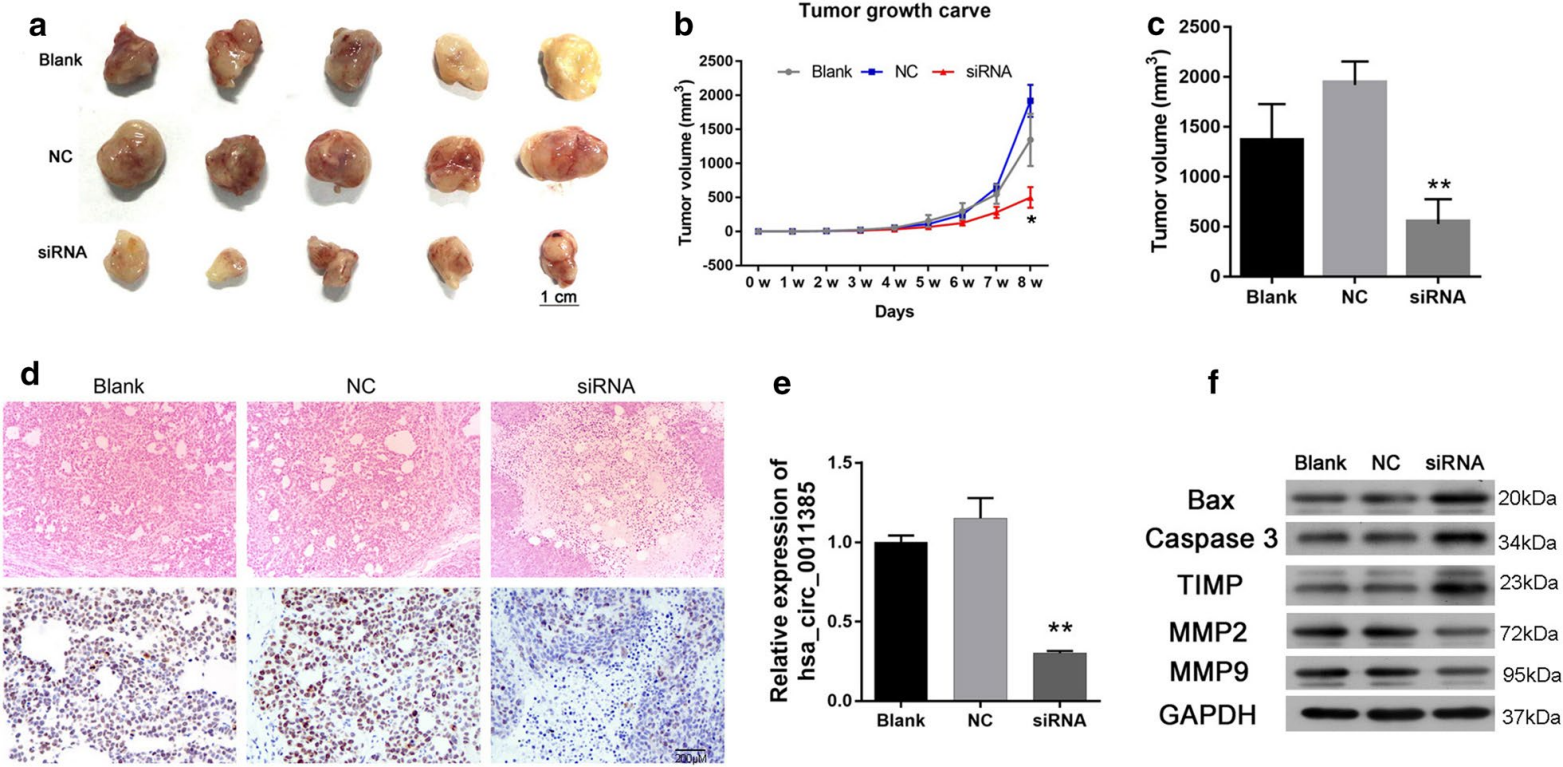
Hsa_circ_0011385 knockdown inhibited thyroid cancer growth in vivo. **a** Xenograft model was established by hypodermic injection of BCPAP cells transfected with hsa_circ_0011385 into BALB/c nude mice. Tumors after 8 weeks post-injection are shown. **b**, **c** The tumor volume and weight were evaluated (*P < 0.05, **P < 0.01 vs. NC group). **d** HE staining was used to assess the pathological conditions of the tumor, and Ki67 expression was detected by IHC assay. Magnification, ×100. Scale bars, 50 µm. **e** Expression of hsa_circ_0011385 was measured by qRT-PCR assay (**P < 0.01 vs. NC group). **f** Bax, caspase-3, TIMP, MMP2 and MMP9 expression levels were evaluated by Western blot assay

Tumor growth was assayed in vivo to further investigate the roles of hsa_circ_0011385. BCPAP cells transfected with hsa_circ_0011385 siRNAs or NC, and the transfection efficiency was detected by qRT-PCR assay. As the data showed that hsa_circ_0011385 siRNAs could reduce hsa_circ_0011385 expression in day 1 to day 4 after transfected (Additional file 1: Figure S1C). The treated BCPAP cells were subcutaneously injected into the left flank of BALB/c nude mice and allowed to grow for 8 weeks. As shown in Fig. 7a, the tumors showed an obvious diminution in the hsa_circ_0011385 siRNA group compared with the NC group. The volume and weight of tumors from hsa_circ_0011385 siRNAs transfected BCPAP cells were significantly reduced compared with tumors from NC transfected cells (*P *< 0.05, *P *< 0.01, Fig. 7b, c). The tumors were also used to detect the pathological conditions and Ki67 expression by using HE staining and IHC assay. HE-stained tumor cells in the blank and NC groups were polygonal, abundant and disordered and had obvious nuclear heteromorphism, more pathological mitosis, less nuclear solids contraction, and smaller area of patchy necrosis; tumor cells in siRNA group were fusiform and round and had dark nuclei, fewer mitotic images and more patchy focal necrosis. IHC assay also indicated that Ki67 expression was reduced in the siRNA group compared to the NC group (Fig. 7d). The efficiency of hsa_circ_0011385 knockdown was also evaluated by qRT-PCR assay. BCPAP cells transfected with hsa_circ_0011385 siRNAs demonstrated a significant downregulation of hsa_circ_0011385compared with cells transfected with NC (*P *< 0.01, Fig. 7e). We then explored whether hsa_circ_0011385 knockdown affected the expression levels of proteins related to apoptosis and metastasis in vivo. The results revealed that downregulation of hsa_circ_0011385 increased the expression levels of Bax, caspase-3 and TIMP, and reduced MMP2 and MMP9 expression levels in BALB/c nude mice with hsa_circ_0011385 siRNAs transfected BCPAP cells (Fig. 7f).

## Discussion

It was well documented that circRNAs were ubiquitously expressed and extremely conserved in all kinds of cells, increasing the complexity of regulatory network of eukaryotic [32]. The majority of circRNAs were originated from the exons of protein-coding genes, and might contain several intron fragments [33]. Under the regulation of various transcriptional regulation factors, protein-coding genes could generate linear or circular RNA molecules through alternative splicing, which is the most critical step in post-transcriptional RNA modification and mature [34]. CircRNAs might become the preferred gene output type when critical spliceosomes or transcriptional regulation factors were dysregulated in cells [35]. Studies indicated that the expression profiles of abnormal circRNAs were revealed to be closely associated with the initiation and progression of various human cancers, including cancer, diabetes, atherosclerotic vascular diseases, systemic diseases, etc. [36–38]. In addition, circRNAs were involved in thyroid cancer [17, 39–41]. In our study, we firstly demonstrated that hsa_circ_0011385 was significantly upregulated in thyroid cancer, and downregulation of hsa_circ_0011385 suppressed thyroid cancer cell proliferation, migration and invasion, and promoted cell cycle arrest and apoptosis.

MiRNAs, characterized by encoding ~ 23 nt RNAs, are a kind of rich ncRNAs without protein-coding capacity [42]. A great number of researches have revealed the critical role of miRNAs in the initiation and progression of diseases [43–45]. Recent researches also showed that miR-361-3p had significant effects in pancreatic ductal adenocarcinoma and non-small cell lung cancer [22, 23]. In our study, we demonstrated that miR-361-3p was negatively correlated with hsa_circ_0011385 in thyroid cancer tissues.

In addition, an extensive body of researches have demonstrated miRNAs could regulate gene expressions by binding to the 3′-untranslated regions (3′-UTR) of the targeted mRNAs to exhibit their posttranscriptional regulatory effects [46, 47]. Current researches also suggested that a number of circRNAs included miRNA response elements (MREs), indicating that circRNAs may serve as miRNAs sponge to reduce the miRNAs levels and releases the targeted genes of miRNAs [48, 49]. For example, circRNA_NEK6 could regulate proliferation and invasion of thyroid cancer by targeting miR-370-3p [50]; circZFR facilitated thyroid cancer cell proliferation and invasion by sponging miR-1261 [51]; circ-ITCH restrained thyroid cancer progression by miR-22-3p [50]. In our study, we proved that hsa_circ_0011385 could negatively regulate miR-361-3p by serving as a sponge, and hsa_circ_0011385 enhanced thyroid cancer cell proliferation, migration and invasion, and inhibited cell cycle arrest and apoptosis by miR-361-3p.

Nowadays, the effect of a special gene or protein on the target disease is often confirmed in in vivo and achieved via using the nude mice. Here, we also used the nude mice to detect the role of hsa_circ_0011385 in the pathological process of thyroid cancer in vivo and demonstrated that down-regulation of hsa_circ_0011385 greatly suppressed the proliferation of thyroid cancer. Wang J et al. found that c-myc siRNA treatment could largely reduce the intimal hyperplasia in vivo [52]. Yan HQ et al. also demonstrated that the number of metastatic tumors were significantly reduced by treating with ATM siRNA [53]. Given the results of these studies, we selected hsa_circ_0011385 siRNAs using for the in vivo experiments. However, many researchers often use the stable knockdown or over-expressed genes or proteins to explore the related role. For example, Wang LQ et al. used the stable overexpressed circular RNA has_circ_0008305 (circPTK2) to explore the influences of it on the NSCLC cell metastasis and demonstrated that circPTK2 overexpression inhibited the metastasis [54]. Therefore, we further need to explore the results of in vivo experiments via using the stable knockdown of hsa_circ_0011385 and confirm the relatively accurate results again.

## Conclusions

The expression of hsa_circ_0011385was increased in thyroid cancer. In vitro, hsa_circ_0011385was shown to promote thyroid cancer cell proliferation, invasion and migration, and to suppress cell cycle progression and apoptosis by negatively regulating miR-361-3p expression. These findings contribute to the understanding of thyroid cancer pathogenesis and suggest a role forhsa_circ_0011385/miR-361-3p axis as a novel therapeutic target in thyroid cancer patients.

## Supplementary information

**Additional file 1: Figure S1.** The Hoechst staining was performed to detect apoptosis and the stability of hsa_circ_0011385 siRNA was verified via real-time PCR. (A and B) The apoptosis ability of BCPAP cells was examined by Hoechst staining, Magnification, 400x. Scale bars, 10 µm. (C) BCPAP cells transfected with hsa_circ_0011385 siRNAs or NC, the transfection efficiency was detected by qRT-PCR assay in day 1 to day 4 after transfected (**P < 0.01 and ***P < 0.001 vs. NC group).

## Data Availability

The datasets used in the current study are available from the corresponding author on reasonable request.
