# Supplementary figures and images for: Hsa_circ_0011385 accelerates the progression of thyroid cancer by targeting miR-361-3p
Source: Cancer Cell Int. 2020 Feb 13;20:49. doi: 10.1186/s12935-020-1120-7 (PMC7017482; doi:10.1186/s12935-020-1120-7)

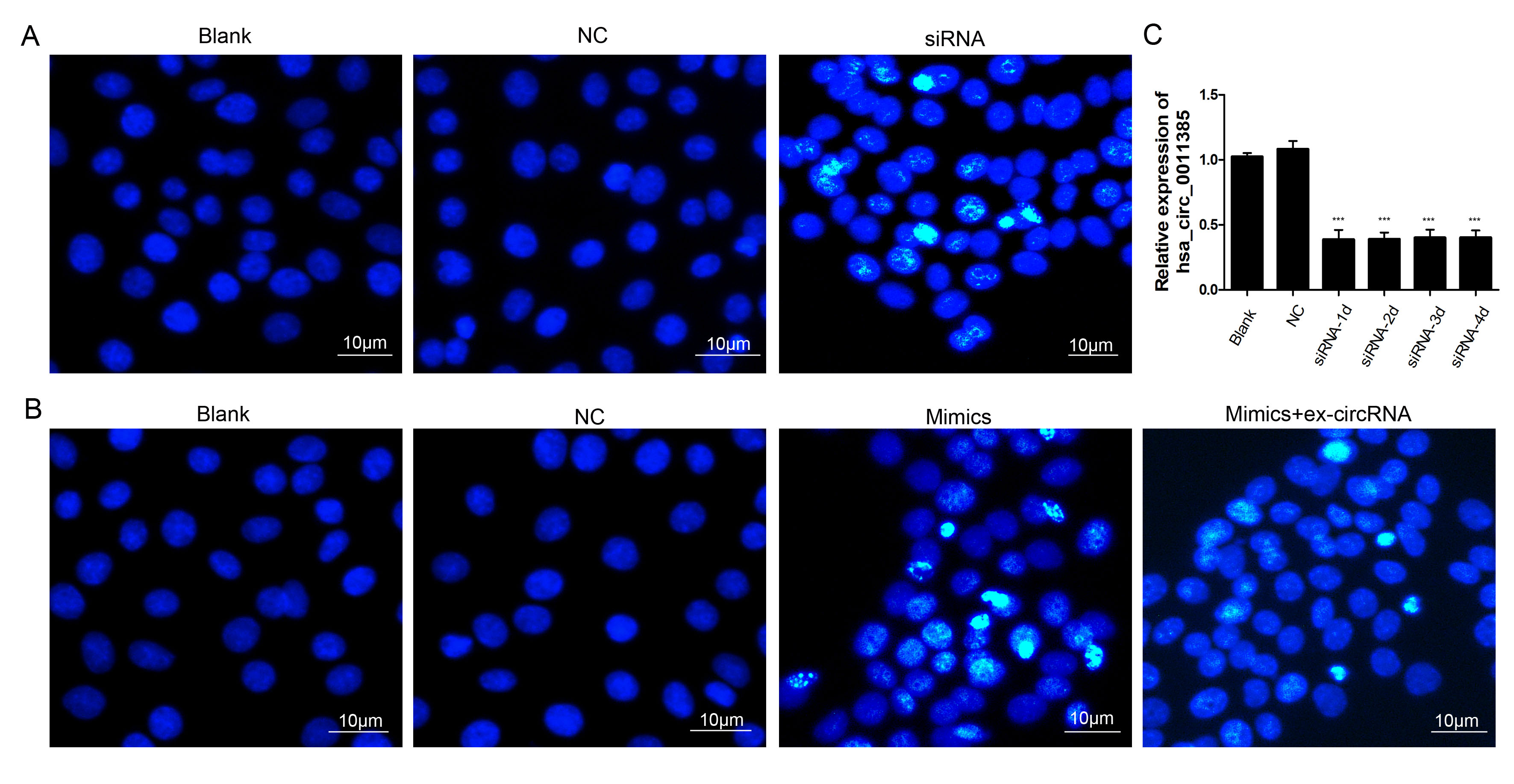

Supplement: Supplementary file 1 — Additional file 1: Figure S1. The Hoechst staining was performed to detect apoptosis and the stability of hsa_circ_0011385 siRNA was verified via real-time PCR. (A and B) The apoptosis ability of BCPAP cells was examined by Hoechst staining, Magnification, 400x. Scale bars, 10 µm. (C) BCPAP cells transfected with hsa_circ_0011385 siRNAs or NC, the transfection efficiency was detected by qRT-PCR assay in day 1 to day 4 after transfected (**P < 0.01 and ***P < 0.001 vs. NC group). [file 12935_2020_1120_MOESM1_ESM.jpg]
